# Impact of Arsenic- and Indium-Terminated InGaAs Stressors on Carrier Confinement, Strain, Defects, and Transport Properties of Tensile-Strained Ge

**DOI:** 10.1021/acsaelm.5c01570

**Published:** 2025-11-04

**Authors:** Sengunthar Karthikeyan, Rishav Khatiwada, Jean J. Heremans, Steven W. Johnston, Ze Zong, Wei Zhou, Mantu K. Hudait

**Affiliations:** † Advanced Devices & Sustainable Energy Laboratory (ADSEL), Bradley Department of Electrical and Computer Engineering, Virginia Tech, Blacksburg, Virginia 24061, United States; ‡ Department of Physics, 1757 Virginia Tech, Blacksburg, Virginia 24061, United States; § 53405 National Renewable Energy Laboratory, Golden, Colorado 80401, United States; ∥ Bradley Department of Electrical and Computer Engineering, Virginia Tech, Blacksburg, Virginia 24061, United States

**Keywords:** germanium, efficient light sources, laser integration, optoelectronics, molecular beam epitaxy, heterostructure

## Abstract

Device-quality tensile-strained Ge (ε-Ge) grown on a large bandgap semiconductor with superior electrical and optical carrier confinement is essential for group-IV-based optoelectronics. Properties of ε-Ge active layers synthesized on In_0.24_Ga_0.76_As buffers with two different surface terminations—arsenic-rich and indium-rich—were experimentally demonstrated, highlighting the factors not considered in theoretical calculations. High-resolution X-ray diffraction and Raman spectroscopy analyses of these ε-Ge/In_0.24_Ga_0.76_As heterostructures confirmed the fully strained (1.6%) and partially relaxed (0.82%) nature of the ε-Ge bonded with arsenic-terminated (Ge_As‑terminated_) and indium-terminated (Ge_In‑terminated_) In_0.24_Ga_0.76_As stressors, respectively. High-resolution cross-sectional transmission electron microscopy showed a coherent, sharp, and fully strained ε-Ge/In_0.24_Ga_0.76_As heterointerface in the Ge_As‑terminated_ heterostructure, whereas microtwin defects were present in the Ge_In‑terminated_ heterostructure. These heterostructures were further characterized by evaluating the minority carrier lifetimes, high for Ge_As‑terminated_ (525 ns) and low for Ge_In‑terminated_ (69 ns), using the photoconductive decay technique. Moreover, band alignment was constructed using X-ray photoelectron spectroscopy, where the Ge_As‑terminated_ heterostructure revealed that both holes and electrons were confined within the ε-Ge active layer as a type-I band alignment with Δ*E*_V, As‑terminated_ = 0.22 eV and Δ*E*_C,As‑terminated_ = 0.38 eV. On the other hand, the Ge_In‑terminated_ heterostructure exhibited a type-II band alignment with Δ*E*_V,In‑terminated_ = – 0.02 eV and Δ*E*_C,In‑terminated_ = 0.53 eV. Furthermore, the magnetotransport properties revealed high mobility (321 cm^2^/(V s)) with single-electron transport in Ge_As‑terminated_ heterostructure and low mobility (3.34 cm^2^/(V s)) with multihole transport in the Ge_In‑terminated_ heterostructure. Therefore, preferring the ε-Ge on the arsenic-rich surface of In_0.24_Ga_0.76_As stressor over the indium-rich surface during material synthesis offers device-quality materials with high carrier lifetime and superior carrier confinement, which can provide an opportunity to fabricate efficient group-IV-based optoelectronic devices.

## Graphical abstract

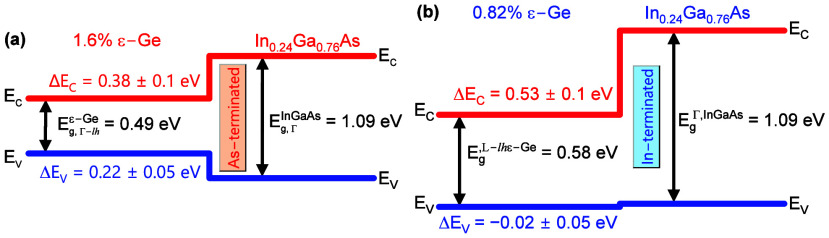

## 1 Introduction

Optoelectronic and photonic devices based on group-IV materials Ge, SiGeSn, and GeSn have continued to be an area of research interest with exceptional recent progress.^1–9^ Of these material systems, germanium (Ge) is favorable for an optically efficient group-IV material^1–4^ due to its pseudodirect bandgap nature, and it is also suitable for electronic applications.^10–13^ The electronic band structure of Ge can be converted to a direct band semiconductor by imparting tensile strain of ∼1.6% ^14,15^ to the Ge (ε-Ge) epilayer using the In_*x*_Ga_1–*x*_As, In_*x*_Al_1–*x*_As, or In_*x*_(GaAl)_1–*x*_As stressor. Moreover, self-assembled tensile-strained Ge quantum dots (QDs), with strain > 1.6%, synthesized on In_*x*_Al_1–*x*_As buffer layers, have also been studied in a three-dimensional approach.^16–18^ These large bandgap stressors acting as “virtual substrates” can also provide superior carrier confinement in the ε-Ge epilayers with large valence band (Δ*E*_V_) and conduction band (Δ*E*_C_) offsets, as a type-I band alignment (i.e., electrons and holes are confined within ε-Ge) is preferred for optoelectronic devices.^1–4,8,9^ However, the experimentally determined band alignment values depend on various factors, such as (i) the bulk-like epitaxial regions of both the group-IV (Ge or ε-Ge) and the groups-III–V (i.e., GaAs, AlAs, InGaAs) materials;^19–21^ (ii) the heterointerfacial stoichiometry, i.e., the bonding of Ge with group-III and group-V atoms;^19–21^ (iii) heterointerfacial charge resulting in an electrostatic potential gradient across the interface;^19–21^ (iv) type and extent of atomic interdiffusion across the heterointerfacial region;^19,22,23^ and (v) defects at the heterointerface.^24^

*In the past*, Pavarelli et al.^25^ addressed (i) and (ii) by evaluating the band offset values using density functional theory for ε-Ge/In_0.30_Ga_0.70_As heterostructure. It has been reported that a type-I band alignment was formed when Ge bonded with 100% group-III atoms at the interface and type-II when Ge bonded with 100% group-V atoms at the interface. However, the band offsets for the intermediate stoichiometries of Ge bonding with mixed group-III and group-V atoms were interpolated. Similarly, Greene-Diniz and Grüning evaluated the band alignment of ε-Ge/In_0.25_Al_0.75_As heterostructure^19^ using first-principles calculations, where interface charges were balanced to achieve a neutral heterointerface using mixed group-IV to groups-III–V bonds. This system exhibited type-I band alignment, when Ge bonded with group-III-terminated or Ge bonded with group-V-terminated InAlAs in an abrupt interface. In addition, they also calculated the impact of atomic interdiffusion on the band alignments and found that arsenic up-diffusion into Ge did not change the type of band alignment, whereas indium up-diffusion into Ge changed the band alignment from type-I to type-II. To date, there is a lack of experimental evidence to correlate the theoretical predictions of the band alignments of group-IV on groups-III–V materials, considering the interfacial bonding of Ge with group-III atoms. However, it has been experimentally demonstrated that defects at the heterointerface can alter the band alignments,^24^ but neither of these theoretical calculations incorporated defects into their models.

This work is the first to demonstrate an experimental investigation on the band alignment of the ε-Ge/In_0.24_Ga_0.76_As heterostructure, where Ge is bonded with different surface-terminated In_0.24_Ga_0.76_As stressor material (arsenic and indium atoms). By the type of InGaAs surface termination during material synthesis (e.g., As- or In-atom-terminated surface), one can alter the heterointerfacial band alignment type and offset values.^19,23,25^ In the present work, ε-Ge/In_0.24_Ga_0.76_As heterostructure material systems, synthesized using solid source molecular beam epitaxy (MBE), were investigated for two types of InGaAs surface terminations during growth: (i) the ε-Ge grown on As-terminated In_0.24_Ga_0.76_As surface and (ii) the ε-Ge grown on In-terminated In_0.24_Ga_0.76_As surface by intentionally maneuvering the heterointerface region with As-rich and In-rich stoichiometry, respectively. These heterostructures were evaluated for their structural, morphological, defect, carrier lifetime, and transport properties to study the material quality of the ε-Ge epilayer. High-resolution X-ray diffraction and Raman spectroscopy confirmed the pseudomorphic nature and the amount of tensile strain in the Ge epilayer. Cross-sectional transmission electron microscopic study was carried out for the defect analysis of these heterostructures. Carrier lifetimes of ε-Ge epilayers were evaluated by using the photoconductive decay technique. In addition, the magnetotransport properties of these heterostructures were evaluated using Hall measurement at 296 and 4.2 K. Furthermore, experimental investigation of the type of band alignment was presented via X-ray photoelectron spectroscopy (XPS) analysis of these ε-Ge/In_0.24_Ga_0.76_As heterostructures to show the impact of surface atom terminations on the band offset values. This work experimentally demonstrates the influence of As- and In-rich ε-Ge/In_0.24_Ga_0.76_As heterointerface engineering on the material properties of tensile-strained Ge that paves the way for the development of group-IV-based optoelectronic applications.

## 2 Experimental Section

### 2.1 Materials Synthesis

Tensile-strained epitaxial Ge layers (ε-Ge)—30 nm (sample S1) and 25 nm (sample S2) thick—were grown on constant composition In_0.24_Ga_0.76_As buffer layers (as the underlying stressors) over (100)/2° semi-insulating GaAs substrates as shown in Figure 1, using a dual chamber solid source MBE system. Since at biaxial tensile strain greater than 1.5%, the Ge transitions to a direct band semiconductor,^15,16^ the 24% In composition was chosen to impart a tensile strain of ∼1.6% to Ge having a lattice constant^23^ of ∼5.658 Å using In_0.24_Ga_0.76_As stressor template (lattice constant^12^ of ∼5.75 Å). Moreover, to realize a virtually defect-free epilayer of ε-Ge, it is essential to limit the thickness below the critical layer thickness (*h*_c_) value, beyond which defects and dislocations would be introduced in the ε-Ge epilayer.^2,4,23,26,27^ Since the critical layer thickness at 1.6% strain is *h*_c_ = 45 nm (according to the People and Bean strain energy balance model^27^), the thicknesses of ε-Ge epilayers in samples S1 and S2 were kept below 45 nm to fully retain the tensile strain from the underlying In_0.24_Ga_0.76_As stressor templates. On the other hand, the 2° offcut in the (100) GaAs substrate toward the [110] direction was chosen to aid efficient strain relaxation of the In_*x*_Ga_1–*x*_As linearly graded buffer (LGB). The atomic surface steps in offcut substrates help the lattice mismatch-induced dislocations in In_*x*_Ga_1–*x*_As LGB to glide along the surface steps and promote uniform strain relaxation in two orthogonal directions.^28,29^ Moreover, metamorphic InGaAs growth on (100) GaAs with 2° misorientation was shown to exhibit better optical properties due to reduced electron trap concentrations than on (100) on-axis GaAs substrate, as reported in ref 30. In addition, to realize a ε-Ge quantum well laser structure, further growth of InGaAs on the top of ε-Ge needs the use of a misoriented substrate, as the surface atomic steps on (100) GaAs with misorientation angle towards the [110] direction helps to avoid the formation of antiphase domains.^31,32^ In this MBE system, the group-IV and groups-III–V growth chambers are interconnected and well-isolated via an ultrahigh-vacuum transfer chamber to avoid interdiffusion of the atomic elements from either side of the chambers during the growth. This also ensures that post III–V growth, the wafer is transferred via ultrahigh vacuum to the group-IV chamber without any exposure to the external environment, avoiding potential oxidation of the InGaAs surface before epitaxial growth of ε-Ge. Prior to the growth of a constant composition In_0.24_Ga_0.76_As buffer, linearly graded buffers of In_*x*_Ga_1–x_As were grown over the homoepitaxial GaAs layer on the (100)/2° GaAs substrates of both samples, S1 and S2. A standard method while synthesizing mismatched heteroepitaxy is to contain the defects and dislocations (due to the lattice mismatch between the GaAs substrate and the constant composition In_0.24_Ga_0.76_As buffer) within the In_*x*_Ga_1–*x*_As LGB.^28–30,32–34^ There are several approaches for designing LGB,^32^ with and without overshoot composition during mismatched heteroepitaxy, to reduce the defects and dislocations in the active layer.^32–34^ In this study, the overshoot graded profile was implemented in the design of the LGB in sample S1, whereas no overshoot composition was utilized for sample S2, as shown in Figure 1. It has been reported that, in the LGB without overshoot, a larger buffer layer thickness is essential for effective strain relaxation than the LGB with overshoot.^33–36^ Therefore, the sample S1 with LGB having 28% In overshoot was grown to be ∼0.69 μm, whereas in the case of sample S2 (without In overshoot), a thicker LGB of 1.25 μm was grown for efficient strain relaxation.

**Figure 1:**
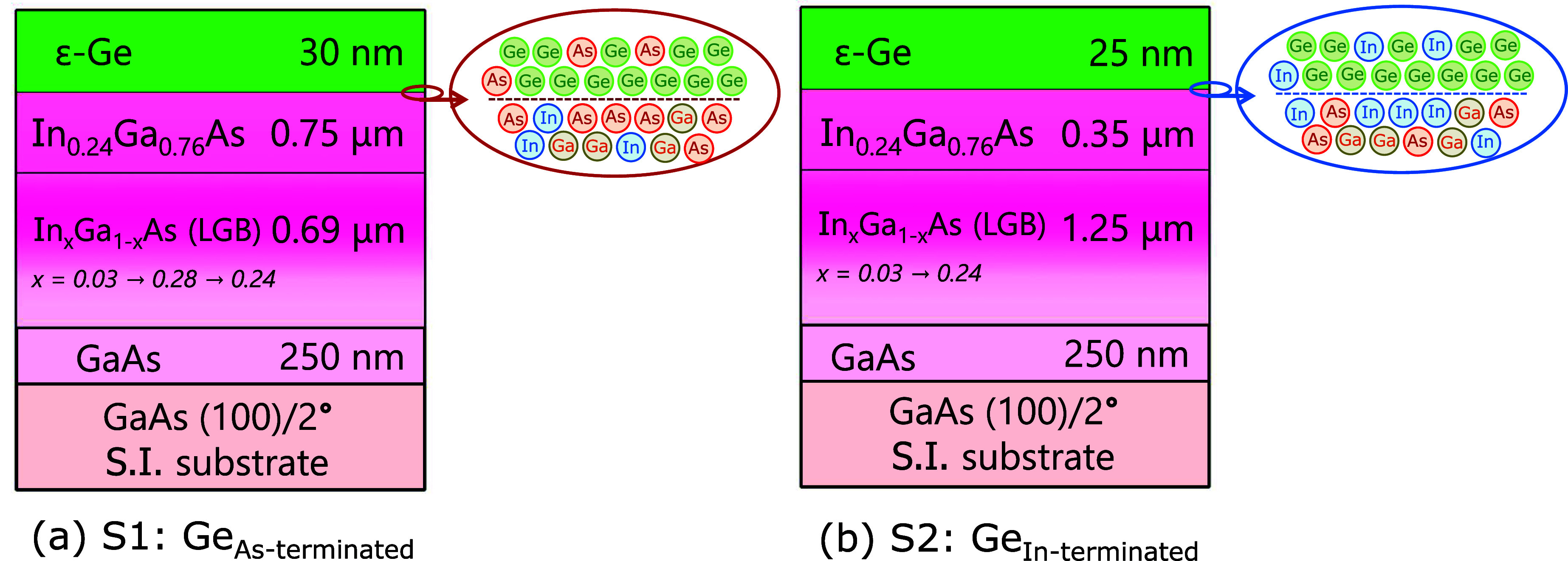
Cross-sectional schematic of (a) 30 nm thick 1.6% ε-Ge on As-terminated (Ge_As‑terminated_) In_0.24_Ga_0.76_As and (b) 25 nm thick 0.82% ε-Ge on In-terminated (Ge_In‑terminated_) In_0.24_Ga_0.76_As heterostructures grown on (100)/2° semi-insulating (S.I.) GaAs substrates.

A reflection high-energy electron diffraction (RHEED) system was used to observe the surface reconstruction during the epitaxial growth as well as oxide desorption of the GaAs substrate at 750 °C^10,31,37^ with arsenic As_2_ overpressure of ∼10^–5^ Torr to balance the volatile escape of arsenic atoms.^10,23,31^ Sharp and streaky (2 × 4) surface reconstruction was observed from the surface of the In_0.24_Ga_0.76_As buffer layer. All of the substrate temperatures reported in this work are thermocouple temperatures, as it is not feasible to measure the real substrate temperatures in our present MBE system due to the lack of pyrometer/band-edge reading capabilities. It has been reported that the difference between the oxide desorption temperature measured by thermocouple (750–800 °C)^37,38^ and the growth temperature of the homoepitaxial GaAs during MBE process is 70–100 °C,^37,38^ also followed in the present work. Whereas the growth temperature of the ε-Ge epilayer was 400 °C, at a growth rate of ∼0.1 Å/s. For sample S1, shown in Figure 1a, after growing the constant composition In_0.24_Ga_0.76_As buffer layer, the sample was cooled to 300 °C with arsenic overpressure and then further down to below 150 °C before transferring the sample to a group-IV chamber via ultrahigh-vacuum transfer chamber at 4 × 10^–10^ Torr, where a 30 nm thick ε-Ge epilayer was grown on the As-terminated In_0.24_Ga_0.76_As buffer layer. In the past, the structural analysis of ε-Ge on As-terminated In_0.24_Ga_0.76_As stressor without using an In overshoot in the In_*x*_Ga_1–*x*_As LGB was reported.^26^ By comparing the reported findings, it is confirmed that the role of the In overshoot in the present work in sample S1 is limited to efficient strain relaxation of In_*x*_Ga_1–*x*_As LGB at lower thickness, without affecting the overlying Ge epilayer in terms of strain, surface morphology, and defect properties. For sample S2, shown in Figure 1b, after growing the constant composition In_0.24_Ga_0.76_As buffer layer, the sample was cooled to 300 °C with arsenic overpressure and then further down to 200 °C with no arsenic overpressure since arsenic atoms do not escape from the surface at this low temperature. At 200 °C temperature, two monolayers (MLs) of indium (In) were grown on In_0.24_Ga_0.76_As buffer layer since previous atom probe tomography (APT) results of ε-Ge/In_0.24_Ga_0.76_As^26^ and Ge/AlAs^39^ heterostructures exhibited arsenic up-diffusion of ∼6 Å (2 MLs) into ε-Ge and Ge epilayers, respectively. Subsequently, a 25 nm thick ε-Ge layer was grown on the In-terminated In_0.24_Ga_0.76_As buffer layer.

### 2.2 Materials Characterization

High-resolution X-ray diffraction (HR-XRD) technique was used for reciprocal space map analysis to determine the crystalline quality and overall structural quality of the semiconductor layers using PANalytical X’Pert Pro X-ray instrument having a pixel and proportional detector with a monochromated Cu Kα (λ = 1.540598 Å) X-ray source. The surface morphology of the ε-Ge/In_0.24_Ga_0.76_As heterostructures was characterized using a Bruker Dimension Icon atomic force microscopy (AFM) equipment operated in the tapping mode, where the surface roughness was determined using the Nanoscope Analysis tool. Minority carrier lifetimes of the ε-Ge epilayers were extracted using the microwave-reflection photoconductive decay (μ-PCD) technique at the National Renewable Energy Laboratory (NREL), where the samples were irradiated from the top with a laser source of 1500 nm excitation wavelength (*E*_ph_ = 0.83 eV) at room temperature. The μ-PCD measurement setup has the sample placed underneath a rectangular waveguide (WR-42; dimensions, ∼4.3 mm × 10.7 mm) that is filled with a 20 GHz microwave signal. Since the surface of the sample is irradiated by a laser source with 10 pulses per second (pulse width = 5 ns) at 8–10 mW power, there is a change in conductivity of the sample due to photogenerated carriers. After waiting for 3 ns to allow the carriers to diffuse away from the surface and deep into the layer, the intensity of the reflected microwave signal is recorded as a μ-PCD signal for 1 μs at 1 ns step size. Raman spectroscopy measurements were performed to calculate the amount of tensile strain using the Alpha 300 RSA+, WITec instrument at 532 nm wavelength, using the 20× objective and 1800 grooves/mm grating at room temperature. Cross-sectional high-resolution transmission electron microscopy (HR-TEM) was used to characterize the quality of the ε-Ge/In_0.24_Ga_0.76_As heterointerface using the JEOL 2100 TEM instrument. Electron transparent foils of thin film cross-section for the TEM imaging were prepared using a Gatan disc grinder to mechanically prethin and polish the sample before ion milling using Gatan PIPS (precision ion polishing system) II. During ion milling, the sample-holding cold stage was maintained at −170 °C to prevent degradation of the sample due to the heat generated by the bombardment of the Ar^+^ ion, and the chamber was kept at ∼4 × 10^–5^ Torr to prevent the redeposition of the milled atomic species back onto the sample. The magnetotransport properties of the material systems at 4.2 K temperature were measured in a ^3^He cryostat where the samples were immersed in liquid ^3^He, and the system has a superconducting magnet, allowing variation of the magnetic field normal to the surface of the sample. Band alignment across the ε-Ge/In_0.24_Ga_0.76_As heterointerface was determined by the X-ray photoelectron spectroscopy technique using a PHI Quantera SXM-03 (scanning XPS microprobe) instrument driven by a monochromatic Al Kα (1486.7 eV) X-ray source. Each sample was cleaned using deionized water for 30 s to remove atmospheric contaminants from the top of the sample surface prior to loading into the XPS chamber. The photoemission spectra emanating from the samples’ surface were collected through a hemispherical electron energy analyzer with a pass energy of 26 eV and at an exit angle of 45° (with respect to the normal to the sample surface). CasaXPS 2.3.25, a software tool, was used to fit the acquired XPS spectral peaks by convolution of Lorentzian and Gaussian mathematical functions,^40^ which aids in identifying the elemental core level (CL) peak positions, where an iterated Sherley background was used to fit the spectral regions representing electron emission due to inelastic scattering. The position of the valence band maximum (VBM) was obtained by linearly fitting the onset of X-ray photoemission spectra from the valence band density of states, which corresponds to an equivalent response from the leading edge of a constant random background noise.^41^ To specifically probe the interfacial region of ε-Ge on In_0.24_Ga_0.76_As in each sample, an Ar^+^ ion gun was used at a low voltage of 1 kV over a 2 mm × 2 mm square region to sputter-etch-reach the interface and acquire the energy distribution curves corresponding to all of the elements—Ge, In, Ga, and As—at the heterointerface. Further sputtering was carried out to collect the XPS spectra from the constant composition In_0.24_Ga_0.76_As buffer layer. An uncertainty for the CL–VBM energy difference was found to be ±0.05 eV, and error analysis yielded a final experimental uncertainty for the measured Δ*E*_V_ as ±0.1 eV.

## 3 Results and Discussion

### 3.1 Structural and Compositional Analysis by X-ray Diffraction

Crystalline quality of both the ε-Ge/InGaAs heterostructure material systems, sample S1, ε-Ge with arsenic-terminated (Ge_As‑terminated_), and sample S2, ε-Ge with indium-terminated (Ge_In‑terminated_) InGaAs buffer, were analyzed using the reciprocal space maps (RSMs), recorded by HR-XRD technique, shown in Figures 2 and 3, respectively. The InAs molar fraction in the linearly graded metamorphic In_*x*_Ga_1–*x*_As buffer layers and their relaxation states were quantitatively determined from the in-plane (a_||_) and the out-of-plane (a_⊥_) lattice constants, calculated using the symmetric (004) and the asymmetric (115) crystallographically orientated RSMs.^42,43^ In the sample S1–Ge_As‑terminated_, the symmetric (004) RLP contour of a constant composition In_0.24_Ga_0.76_As buffer layer lying below the GaAs substrate, in Figure 2a, confirmed that it has a larger *a*_⊥_. The indium (In) overshoot within the In_*x*_Ga_1–*x*_As LGB, which has an RLP contour below the constant composition In_0.24_Ga_0.76_As, facilitates efficient strain relaxation during metamorphic In_*x*_Ga_1–*x*_As growth over a GaAs substrate.^32–36^ Symmetrical *Q*_*x*_–*Q*_*z*_ spread of the RLP contours indicates that the metamorphic layers have uniform crystallinity with less mosaicity in the in-plane direction and uniformity of the out-of-plane lattice constant in the growth direction. The (004) RLP of the ε-Ge epilayer lying above the GaAs substrate and the In_0.24_Ga_0.76_As stressor template indicates that it has a smaller *a*_⊥_ than both the GaAs substrate and the constant composition In_0.24_Ga_0.76_As buffer layer. The ε-Ge epilayer, being only 30 nm thin, has a low-intensity contour; however, it has a symmetric *Q*_*x*_–*Q*_*z*_ spread, indicating a good crystalline quality of the ε-Ge epilayer. The asymmetric (115) RSM of the S1-Ge_As‑terminated_ sample, shown in Figure 2b, is essential to confirm that the tensile strain in the Ge epilayer is retained to maintain the registry of atoms with the underlying In_0.24_Ga_0.76_As stressor. Using the in-plane and out-of-plane lattice parameters calculated from the symmetric (004) and asymmetric (115) RSMs, and employing the methods reported in refs 42 and 43 , the InAs molar fraction was calculated to be ∼24%. The *Q*_*x*_ alignment of the centroids of the RLP contours of ε-Ge epilayer and the constant composition In_0.24_Ga_0.76_As buffer layer shows that there is no relaxation of the 1.6% in-plane tensile strain (ε_||_) in the ε-Ge epilayer and hence no relaxation-induced tilt,^42,43^ whereas 869 arcsecs of relaxation-induced tilt was noted from the constant composition In_0.24_Ga_0.76_As layer, and no such tilt was noted in ε-Ge epilayer, confirming that the 30 nm thick ε-Ge is below the critical layer thickness^26,27^ beyond which nucleation of misfit dislocations (MDs) and threading dislocations (TDs) introduce defects.^2,4,23,26,27^ In our previous work, a ε-Ge/In_0.24_Ga_0.76_As heterostructure^26^ was demonstrated using a In_*x*_Ga_1–*x*_As LGB design (*x* = 0.03–0.24) without using an In overshoot, wherein^26^ similar structural characteristics were obtained from the X-ray analysis of the In_0.24_Ga_0.76_As stressor template and the ε-Ge epilayer with 1.6% tensile strain as the sample S1-Ge_As‑terminated_ that has an In overshoot incorporated in the In_*x*_Ga_1–*x*_As LGB layer.

**Figure 2:**
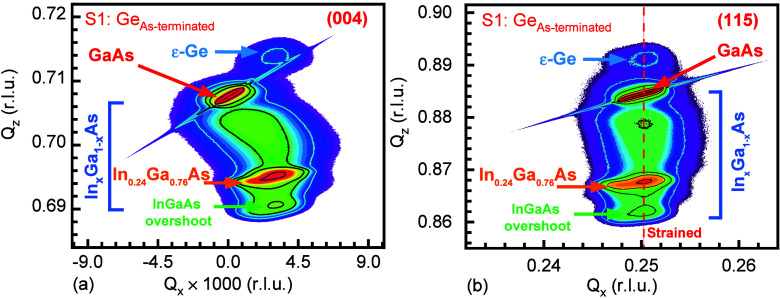
High-resolution reciprocal space maps (RSMs) of S1: 1.6% ε-Ge/As-terminated-In_0.24_Ga_0.76_As taken along the (a) symmetric (004) and (b) asymmetric (115) crystallographic orientations (r.l.u. is reciprocal lattice unit). Alignment of ε-Ge and In_0.24_Ga_0.76_As reciprocal lattice point contours along the (115) orientation indicates a fully tensile-strained Ge epilayer.

**Figure 3:**
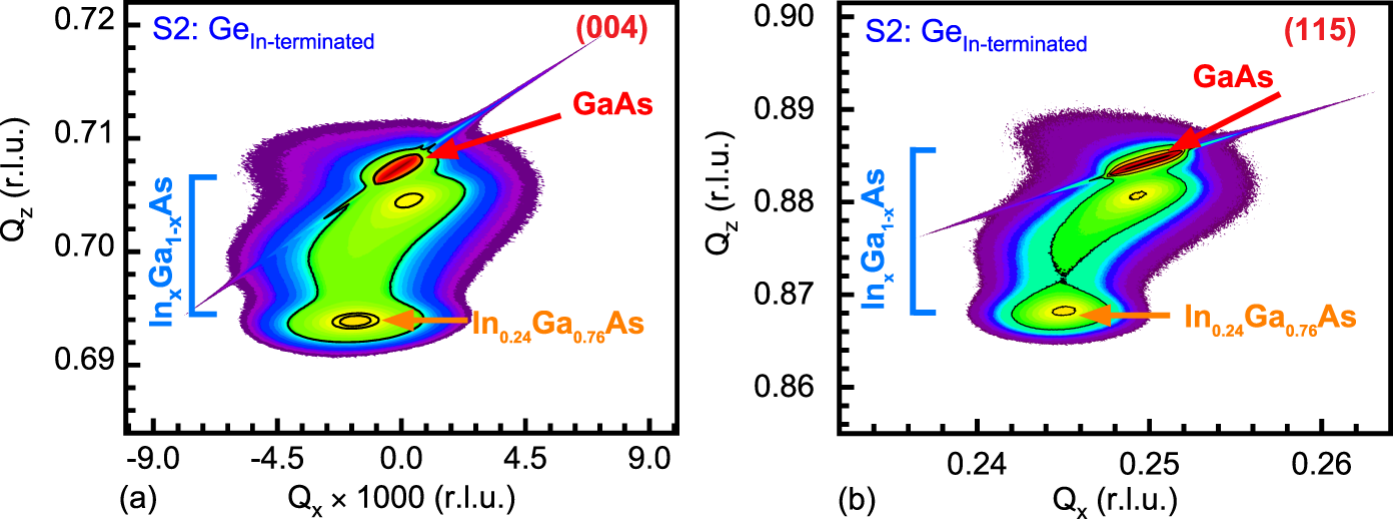
High-resolution reciprocal space maps (RSMs) of S2: 0.82% ε-Ge/In-terminated-In_0.24_Ga_0.76_As taken along the (a) symmetric (004) and (b) asymmetric (115) crystallographic orientations. Absence of a ε-Ge RLP contour indicates that the nature of the ε-Ge epilayer is less crystalline.

In the case of sample S2-Ge_In‑terminated_, the symmetric (004) RSM in Figure 3a shows similar positions of the RLP contour diffracted from the constant composition In_0.24_Ga_0.76_As buffer layer. Note that there was no overshoot of the In composition incorporated into the linearly graded In_*x*_Ga_1–*x*_As buffer layer. However, the efficient strain relaxation of the In_*x*_Ga_1–*x*_As LGB layer was aided by a 1.25 μm thick layer growth. Similar *Q*_*x*_–*Q*_*z*_ symmetricity of the InGaAs RLP contour affirms mosaicity-free uniform crystalline quality of the constant composition In_0.24_Ga_0.76_As buffer layer. Notably, there is no relation between the 2 MLs of In termination and the *Q*_*x*_ peak position of the In_0.24_Ga_0.76_As RLP contour in both the (004) and (115) RSMs shown in Figure 3. Strain-induced relaxation in the In_*x*_Ga_1–*x*_As LGB introduces tilt in the buffer layers^26,28–30,42,43^ that is observable in different amounts based on the angle of azimuth (Φ) at which the X-ray is incident on the substrate and diffracted to the detector. This creates an apparent difference in the relative positions of In_0.24_Ga_0.76_As and GaAs RLPs when both samples S1 and S2 are compared to each other with respect to both symmetric (004) and asymmetric (115) reflections in the RSM analysis. Moreover, there is no visible RLP contour of the ε-Ge epilayer, either along the symmetric (004) crystallographic orientation, shown in Figure 3a, or along the asymmetric (115) orientation, shown in Figure 3b. Since the X-ray analysis of a semiconductor needs the material to be highly crystalline to record the RLP,^44^ the absence of ε-Ge RLP could be attributed to the lack of its uniform crystalline nature or the presence of defects, which would be investigated by cross-sectional TEM analysis.

### 3.2 Surface Morphology by Atomic Force Microscopy

Surface topography of a metamorphic or a pseudomorphic epitaxial layer in a heterostructure material system facilitates qualitative characterization of the isotropic or anisotropic relaxation of the layers and quantitatively determines the surface roughness.^23,26,36^ The AFM technique helps to characterize the surface morphology quantitatively, with the root-mean-square roughness (*R*_q_) value being the key metric for the surface of a semiconductor layer. Moreover, it qualitatively reveals the uniformity as well as the directional nature of strain relaxation in metamorphic heteroepitaxy, as observed from the surface cross-hatch pattern. In a metamorphic heteroepitaxy, the strain-relieving mechanism within the graded buffer layer occurs through the nucleation of TDs in the bulk regions and the MDs at the heterointerface.^2,4,28–30,32^

In a zinc blende semiconductor with (100) orientation, the dislocations thread to the top epilayers through the {111} slip planes by lateral propagation along the <110> directions, referred to as α and β dislocations, that are orthogonal to each other.^26,28–30^ The extra half-plane of atoms (positive or negative dislocations) between the {111} layers could belong to either the shuffle or the glide sets^32^—the shuffle set has the extra half-plane between widely spaced {111} planes, whereas the glide set has it between the narrowly spaced {111} planes. Both in the zinc blende and the diamond structures, the MDs corresponding to the glide sets are energetically favorable.^26,32^ If we consider GaAs, both the α- and β-type dislocations could have either As or Ga atoms terminated at their cores; (i) α dislocations have Ga atoms in the shuffle set and As atoms in the glide set; (ii) β dislocations have As atoms in the shuffle set and Ga atoms in the glide set. In short, the α dislocations observed in the III–V epitaxial layers have group-V atom terminations at their core, whereas β dislocations have group-III atom terminations at their core; i.e., both the dislocations belong to the glide sets. The dislocation velocities of α and β dislocations are disproportionate, with the α dislocations found to be more glissile and nucleating first along the [1 1̅ 0] direction in the In_*x*_Ga_1–*x*_As graded buffer, followed by the β dislocations nucleating along the [1 1 0] direction in the later stages of continued thick buffer layer growth during heteroepitaxy.^26^

The AFM micrographs of 20 × 20 μm^2^ regions from samples S1-Ge_As‑terminated_ and S2-Ge_In‑terminated_, shown in Figure 4, display the cross-hatch patterns replicated in the top layer of Ge epitaxy carried over from the underlying InGaAs buffer, where both the α and β dislocation lines are observed. The overall RMS roughness in the 20 × 20 μm^2^ regions was determined to be *R*_q,As‑terminated_ = ∼5.47 nm and *R*_q,In‑terminated_ = ∼6.14 nm. To determine the distribution of strain relieved in the orthogonal α and β directions, the line profiles across 15 μm lengths were extracted, as shown in Figure 4. The α dislocations have shorter peaks to valleys than the β dislocations in both samples, indicating that there is a greater strain relaxation of the InGaAs buffer along the [1 1̅ 0] than the [1 1 0] direction. The *R*_q_ values along the [1 1̅ 0] direction (α dislocation) for S1-Ge_As‑terminated_ is ∼3 nm and S2-Ge_In‑terminated_ is ∼4.4 nm, whereas along the [1 1 0] direction (β dislocation) for S1-Ge_As‑terminated_ is ∼5.56 nm and S2-Ge_In‑terminated_ is ∼6.57 nm. However, there is less irregularity (i.e., surface undulations) in the peaks and valleys from the surface of S1-Ge_As‑terminated_ than in the S2-Ge_In‑terminated_ sample, even though their extracted *R*_q_ values do not have significant differences. Previously reported^26^ surface morphology of the 1.6% ε-Ge epilayer synthesized on As-terminated In_0.24_Ga_0.76_As stressor without In overshoot in the In_*x*_Ga_1–*x*_As LGB also exhibited a uniform cross-hatch pattern similar to that of sample S1 that has the In overshoot incorporated in the In_*x*_Ga_1–*x*_As LGB. To understand the surface morphological impact of As-terminated and In-terminated In_0.24_Ga_0.76_As buffer layers on the ε-Ge epilayer, the minority carrier lifetime values were extracted using the μ-PCD technique, as presented below.

**Figure 4:**
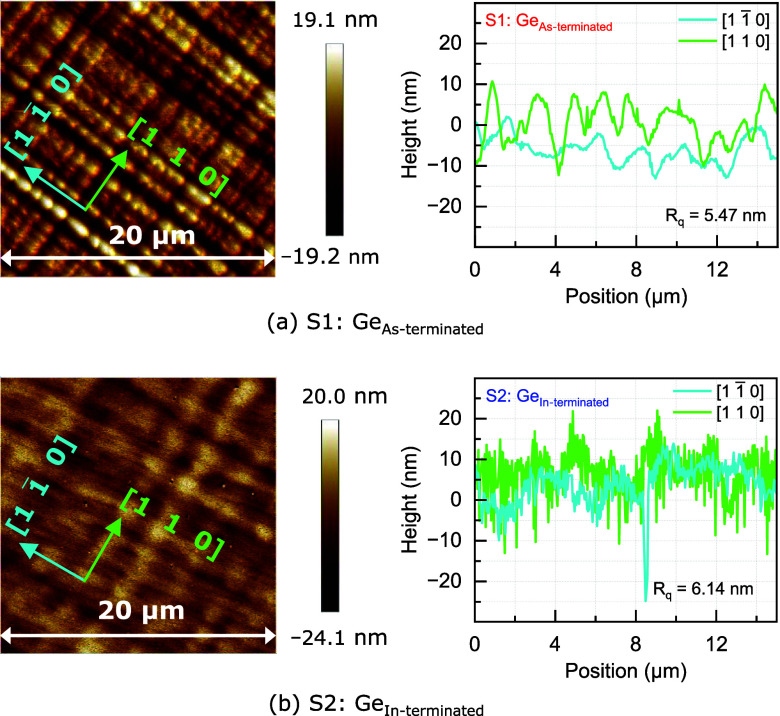
AFM micrographs and corresponding line profiles from the surface of ε-Ge grown on (a) S1: As-terminated, and (b) S2: In-terminated In_0.24_Ga_0.76_As layers, demonstrating RMS roughnesses of 5.47 and 6.14 nm over the 20 × 20 μm^2^ scan area. More uniform and smooth surface morphology was observed on S1-Ge_As‑terminated_ than on the S2-Ge_In‑terminated_ sample, as evident from the line profiles.

### 3.3 Minority Carrier Lifetime by μ-PCD Technique

Evaluation of the minority carrier lifetime of a semiconductor material is effective in characterizing the quality of the material.^45–49^ There are numerous techniques available to determine the carrier lifetime, such as transient free carrier absorption, resonant coupled photoconductive decay, μ-PCD, time-resolved photoluminescence, etc.^48–50^ Here, the minority carrier lifetimes were extracted from the μ-PCD decay signal recorded by using a laser source of 1500 nm excitation wavelength (*E*_ph_ = 0.83 eV) at 300 K, shown in Figure 5. A wavelength of 1500 nm excitation was used to excite the carriers in both the ε-Ge epilayers, as its energy band gap in S1-Ge_As‑terminated_ is 0.49 eV^2–4,15^ and S2-Ge_In‑terminated_ is 0.58 eV.^2–4,15^ At this wavelength, the electrons are excited from the valence band to both the *L*- and Γ-valleys of the conduction band in the ε-Ge epilayer. The initial peak around the 100 ns position corresponds to the photogenerated carriers near the top surface of each ε-Ge epilayer. Faster decay in this region is attributed to the surface state-induced recombination of the photogenerated carriers. The remaining carriers continue to diffuse deep into the ε-Ge epitaxial layer away from the surface. The minority carrier lifetimes were extracted by fitting this curve to a single exponential decay function $V_{\mu \mathrm{‐PCD}}\left(t\right)=V_{o}e^{-t/\tau_{m}}$ , where *V*_o_ is the peak signal intensity and *τ*_m_ is the minority carrier lifetime after the initial surface recombination induced decay.^45^ The μ-PCD minority carrier lifetime of the ε-Ge epilayer in the S1-Ge_As‑terminated_ sample was extracted to be ∼525 ns, whereas for the S2-Ge_In‑terminated_ sample it was ∼69 ns. Though there is an insignificant difference between the RMS roughnesses of both samples’ surfaces, minority carrier lifetime is impacted by more surface undulations in the S2-Ge_In‑terminated_ sample, as discussed above by AFM, leading to more carriers lost to the surface-state-induced recombination than in the S1-Ge_As‑terminated_ sample. A higher surface recombination leads to a faster diffusion rate of the remaining carriers deep into the ε-Ge epitaxial layer away from the surface, contributing to a lower carrier lifetime. Evident from the μ-PCD signal around the 350 ns time scale, the drop in the signal intensity for the S1-Ge_As‑terminated_ sample is less than an order of magnitude compared to 3 orders of magnitude for the S2-Ge_In‑terminated_ sample from the 100 ns time scale. This faster decay/recombination in the epitaxial region away from the surface could be attributed to more defects and dislocations in the ε-Ge epilayer of the S2-Ge_In‑terminated_ sample, as presented by the amount of strain relaxation using Raman spectroscopy and the defect analysis using HR-TEM in the next sections.

**Figure 5:**
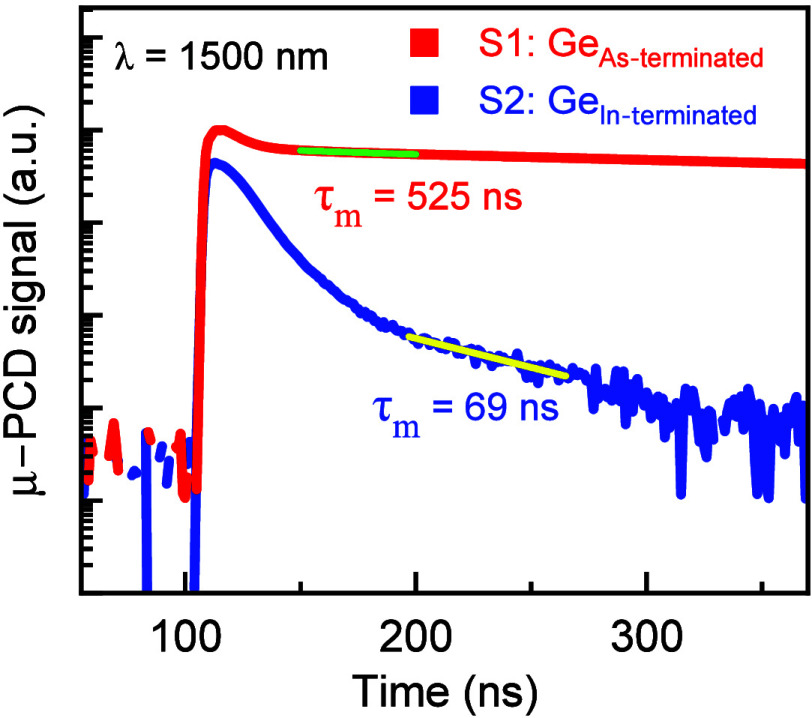
μ-PCD signal versus time acquired at 300 K with 1500 nm excitation wavelength from the ε-Ge epilayers in S1-Ge_As–terminated_ and S2-Ge_In–terminated_ samples, with the respective minority carrier lifetimes (*τ*_m_) of 525 and 69 ns extracted by fitting the curve to an exponential decay after the initial surface recombination induced decay.

### 3.4 Strain Analysis by Raman Spectroscopy

Raman spectroscopy is a standard characterization technique used to study the lattice dynamics, structural, and vibrational properties of a material via the phononic oscillations in the material.^51,52^ Differences in the incident and the scattered frequencies of the photonic signals (laser), due to Stokes–anti-Stokes scattering, give rise to unique Raman wavenumber peaks for each element in the material. This technique has been widely used in the semiconductor industry to determine the compositional elements and the amount of strain in the semiconductor layers of interest in the heterostructures.^23,26,36,51,53^ In the present work, Raman measurements (of Stokes scattering) were carried out in the backscattering geometry,^52^ where the vibrational modes acquired for the (100) oriented semiconductors arise only from the long wavelength (*k* = 0) Ge–Ge longitudinal optical (LO) phonon modes. The deformation potential due to tetragonal distortion in the (100) backscattering Raman spectroscopy splits the 3-fold degeneracy (of phonons) at the Brillouin zone center (*k* = 0) into a singlet mode and a doublet mode. The observed singlet mode (LO phonons) corresponds to the lattice displacement perpendicular to the interface (i.e., growth direction), whereas the doublet mode (TO phonons) corresponds to the displacement parallel to the interface, and it is not observable in the (100) backscattering geometry. The in-plane strain is calculated using the relation Δω = *b*ε_||_ cm^–1^,^51,52^ where *b* is a material parameter representing the phonon deformation potentials, compliance tensor elements, and the zero-strain Raman shift (ω_0_) of the material. Here, the Raman shift recorded from a bulk n-type Ge (doping concentration of 6 × 10^16^ cm^–3^) sample as reference, having zero-strain or tetragonal distortion, was noted to be ω_0_ = 300.38 ± 0.01 cm^–1^ at room temperature,^51^ and the *b* value for Ge is −415 cm^–1^.^53^ Raman spectra recorded from the sample S1-Ge_As‑terminated_ shown in Figure 6, exhibited Raman wavenumbers of 293.87 cm^–1^ giving Δω = −6.51 cm^–1^ and a strain value of ε_||_ = 1.57%. Notably, the strain imparted by the As-terminated In_0.24_Ga_0.76_As stressor synthesized using an In_*x*_Ga_1–*x*_As LGB design without an In overshoot was also 1.57%,^26^ in agreement with the strain imparted to Ge epilayer in sample S1 that has In_*x*_Ga_1–*x*_As LGB with In overshoot. However, the Raman spectra recorded from sample S2-Ge_In‑terminated_, shown in Figure 6, exhibited Raman wavenumbers of 296.99 cm^–1^ giving Δω = −3.39 cm^–1^ with corresponding strain values of ε_||_ = 0.82%. The Raman strain value of the S1-Ge_As‑terminated_ sample agrees closely with the strain value (1.6%) determined by X-ray analysis, affirming that the Ge epilayer remains strained in registry with the underlying As-terminated In_0.24_Ga_0.76_As stressor template, whereas the Ge epilayer in the S2-Ge_In‑terminated_ sample relaxed to 0.82% tensile strain, not in registry with the underlying In-terminated In_0.24_Ga_0.76_As stressor template. Further investigation was carried out to identify the strain relaxation mechanism using TEM analysis, discussed below.

**Figure 6:**
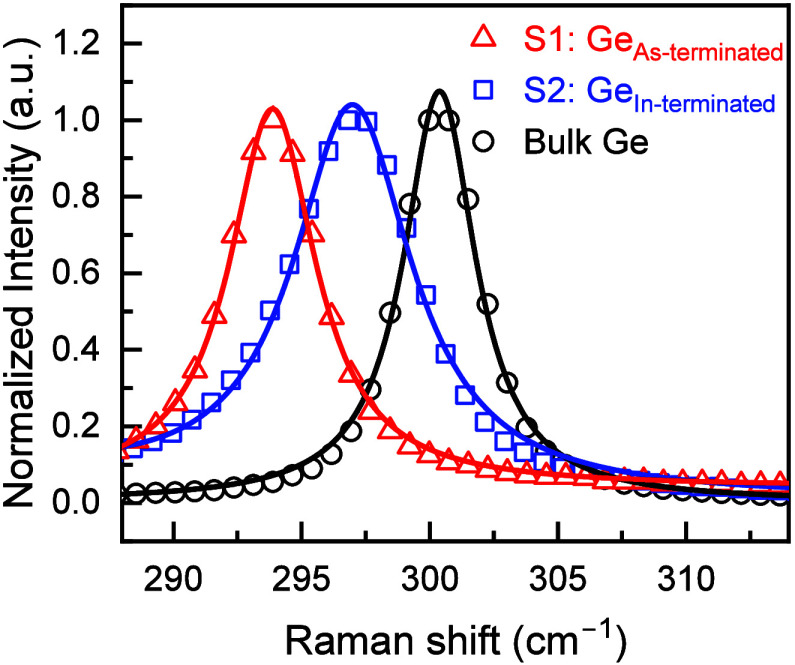
Raman spectra recorded in the (100) backscattering geometry from the ε-Ge epilayers of S1-Ge_As‑terminated_ and S2-Ge_In‑terminated_ samples, grown on As-terminated and In-terminated constant composition In_0.24_Ga_0.76_As buffer layers depicting Ge–Ge LO phonon mode Raman wavenumbers. Tensile strain in the Ge epilayer grown on In-terminated In_0.24_Ga_0.76_As buffer relaxed to almost half the amount, 0.82%, in comparison to the fully strained Ge, 1.57%, on As-terminated In_0.24_Ga_0.76_As buffer.

### 3.5 Defect Analysis by TEM

Investigation of the material quality and characteristic nature of the ε-Ge/In_0.24_Ga_0.76_As heterointerface region in each of the samples S1-Ge_As‑terminated_ and S2-Ge_In‑terminated_ was carried out using cross-sectional HR-TEM analysis. Panels a–e of Figure 7 and panels a–f of Figure 8 show the TEM micrographs and related defect analysis using the fast Fourier transform (FFT) and inverse-FFT patterns from the HR-TEM image of the heterointerface region along the [011] direction. Figure 7a (Figure 8a) depicts the low-resolution TEM (LR-TEM) micrograph of the S1-Ge_As‑terminated_ (S2-Ge_In‑terminated_) heterostructure material system with each layer marked, namely, the GaAs substrate, the In_*x*_Ga_1–*x*_As LGB, and the constant composition In_0.24_Ga_0.76_As buffer layer. The LR-TEM micrographs show that the defects (MDs and TDs) arising due to the lattice mismatch between the constant composition In_0.24_Ga_0.76_As buffer layer and the GaAs substrate are confined within the In_*x*_Ga_1–*x*_As LGB layer. There is no apparent propagation of the TDs and MDs visible from the LGB into the constant composition In_0.24_Ga_0.76_As layer, thereby aiding the growth of a ε-Ge epilayer on a defect mitigated constant composition In_0.24_Ga_0.76_As virtual substrate as the stressor layer. Figure 7b shows the HR-TEM micrograph of the ε-Ge/In_0.24_Ga_0.76_As heterointerface in the S1-Ge_As–Terminated_ sample where the ε-Ge epilayer retains the in-plane lattice arrangement from the underlying In_0.24_Ga_0.76_As layer, with the inset showing visible negative and positive edge dislocations only in In_0.24_Ga_0.76_As buffer along the (1̅ 1̅ 1) plane annihilating each other. Note that this dislocation is ∼5 nm away from the heterointerface region, thereby maintaining the pristine quality of the heterointerface. In a compressively strained epitaxial layer, the 30° partial dislocation nucleates first followed by the 90° partial dislocation in the (100) zinc blende semiconductor heteroepitaxy to allow the strain relaxation by formation of 60° misfit dislocation beyond the critical layer thickness as the end result.^26,32,54^ Also, it is observed in the relaxed constant composition In_0.24_Ga_0.76_As buffer layer that the negative edge 90° dislocation is canceled out by a positive edge 90° dislocation as shown in the inset of Figure 7b. Note that the inset is a representation of a few such mitigations of the dislocations in the In_0.24_Ga_0.76_As epilayer away from the interfacial region. Figure 7c is the FFT pattern of the HR-TEM in Figure 7b, which represents the diffraction pattern of the cross-section captured and indexed along the [011] direction (zone axis).^55^ The indexed diffraction spots of the slip planes (1̅ 1 1̅) and (1̅ 1̅ 1) were masked to obtain their respective inverse-FFT patterns in Figure 7d,e, respectively. It is evident that the (1̅ 1 1̅) plane does not have an extra half-plane of atoms, representing the edge misfit dislocations, whereas the (1̅ 1̅ 1) plane has an extra half-plane of atoms in both directions (positive and negative), thereby mitigating the dislocation in the In_0.24_Ga_0.76_As buffer layer. However, no such misfit dislocations were noted in the ε-Ge epilayer, showing superior quality and thereby retaining the in-plane tensile strain from the underlying In_0.24_Ga_0.76_As layer. Previously reported^26^ HR-TEM analysis of a 1.6% ε-Ge epilayer on As-terminated In_0.24_Ga_0.76_As stressor without using an In overshoot in the In_*x*_Ga_1–*x*_As LGB design exhibited a superior crystalline nature of the ε-Ge epilayer. This further supports that irrespective of whether the LGB design has an In overshoot or no overshoot, it has minimal or no impact on the structural quality of the ε-Ge epilayer over the constant composition In_0.24_Ga_0.76_As stressor layer. Hence, the ε-Ge epilayer was proven to be of device quality with no apparent propagation of the threading or misfit dislocations into the active region, aided by the virtual InGaAs substrate.

**Figure 7:**
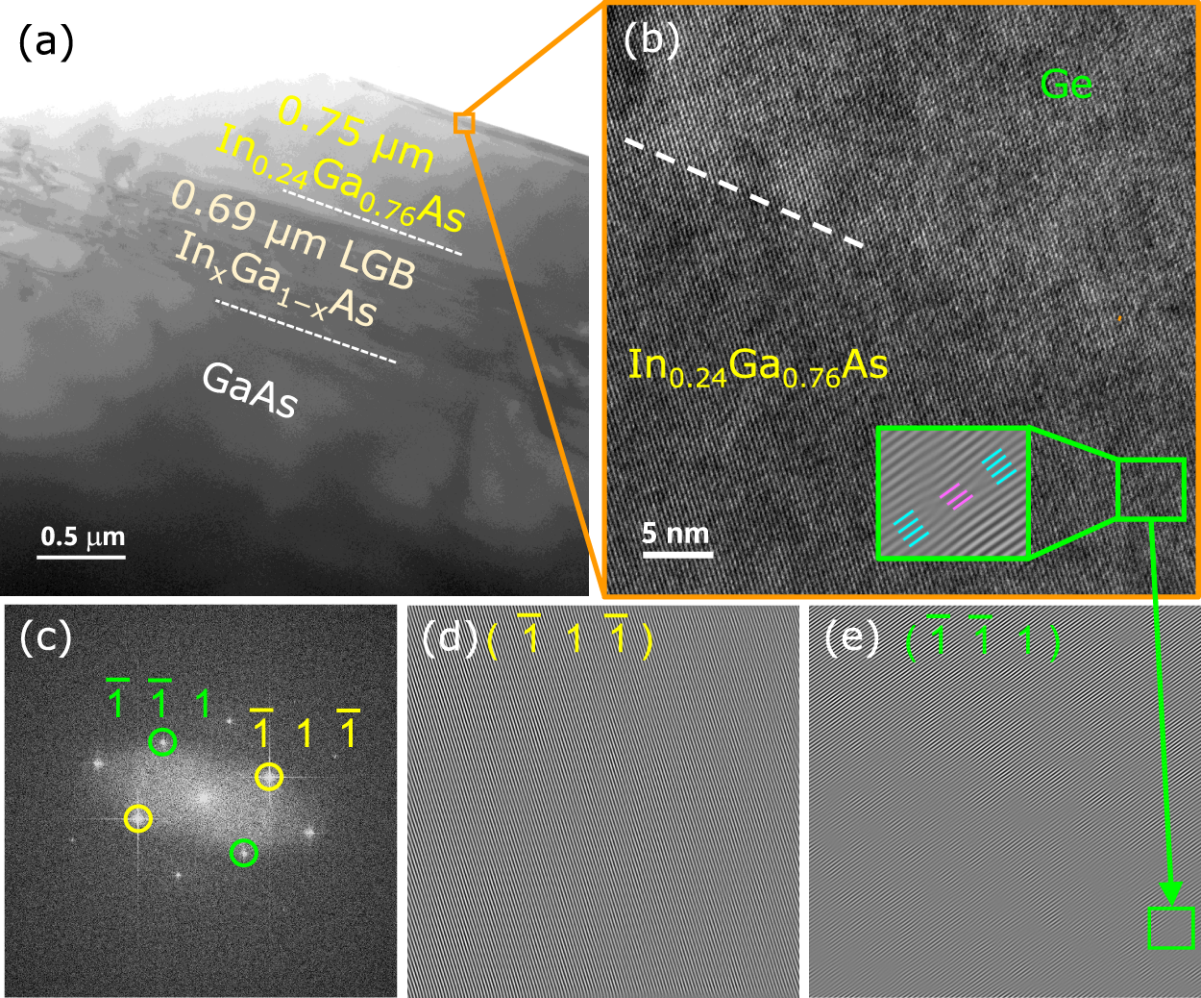
(a) Low-resolution cross-sectional TEM micrograph of the 1.6% ε-Ge on As-terminated In_0.24_Ga_0.76_As on linearly graded In_*x*_Ga_1–*x*_As buffer over (100)/2° semi-insulating GaAs substrate. (b) High-resolution micrograph of the ε-Ge/In_0.24_Ga_0.76_As pristine heterointerface and fully strained Ge epilayer, with the inset displaying the In_0.24_Ga_0.76_As region far away from the interface where negative and positive misfit dislocations annihilate each other in the (1̅ 1̅ 1) crystallographic plane. (c) Fast Fourier transform (FFT) pattern of HR-TEM micrograph in panel b. (d) Inverse-FFT of the (1̅ 1 1̅) plane showing no dislocations; (e) (1̅ 1̅ 1) plane in In_0.24_Ga_0.76_As showing misfit dislocations {inset in panel b}.

**Figure 8:**
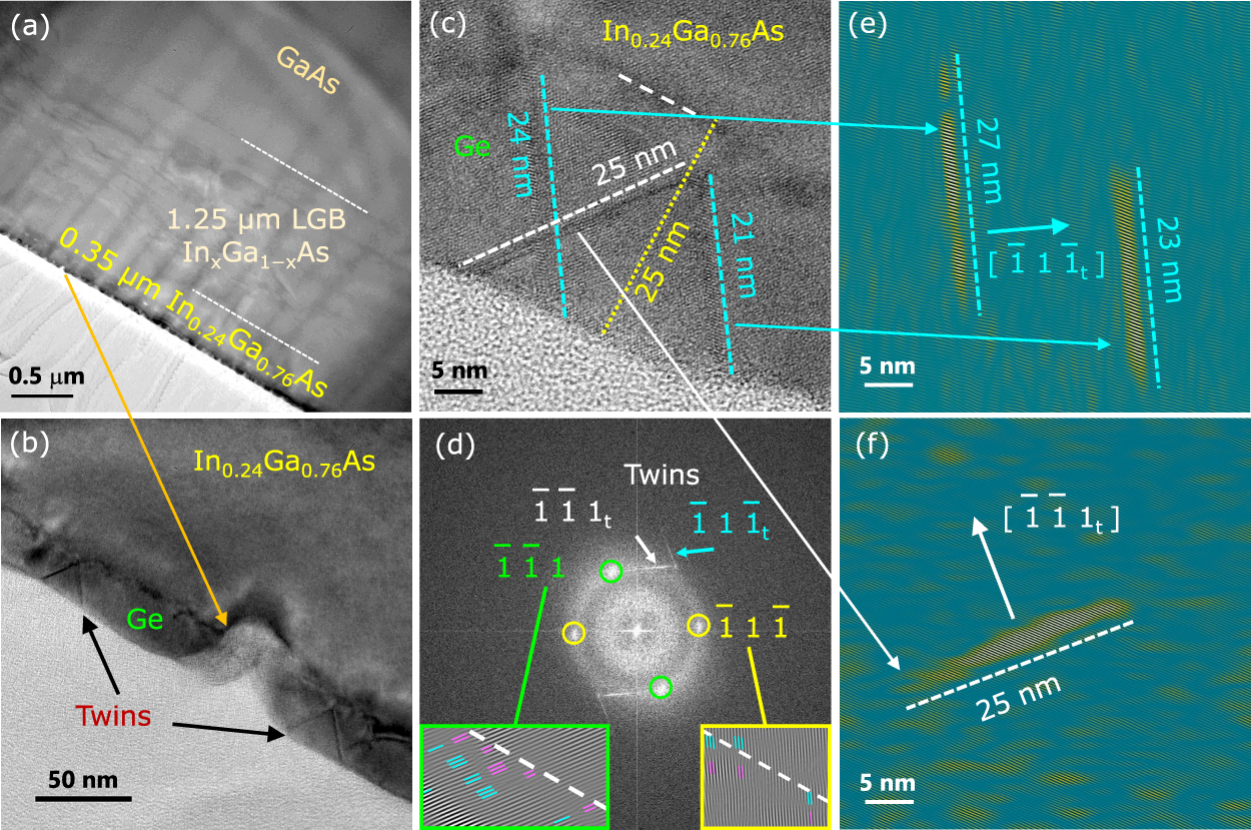
(a) Cross-sectional LR-TEM micrograph of the 0.82% ε-Ge on In-terminated In_0.24_Ga_0.76_As on linearly graded In_*x*_Ga_1–*x*_As buffer over (100)/2° semi-insulating GaAs substrate. (b) Twin defects in the ε-Ge epilayer with uncoalesced regions in between. (c) High-resolution micrograph of the ε-Ge/In_0.24_Ga_0.76_As heterointerface, with the twin defects apparently originating at regions ∼4 nm from the heterointerface. (d) FFT pattern of HR-TEM micrograph in panel c, with twin defects indexed: inset displaying (1̅ 1̅ 1) plane with positive and negative edge dislocations and (1̅ 1 1̅) plane showing negative edge dislocations from the heterointerface. (e) Inverse-FFT of (1̅ 1 1̅_t_), twin (sharp, intense) defects initiating at the heterointerface. (f) Inverse-FFT of (1̅ 1̅ 1_t_), twin (less sharp, less intense) defects possibly initiating at the heterointerface.

From the LR-TEM micrograph of the S2-Ge_In‑terminated_ sample in Figure 8a, the top ε-Ge epilayer grown on an In-terminated constant composition In_0.24_Ga_0.76_As layer shows twin defects as well as certain regions of uncoalesced Ge epilayer across the sample. A representative image is portrayed in Figure 8b, where the targeted 18 nm growth of ε-Ge epilayer resulted in ε-Ge regions of varying thickness from 21 to 25 nm in the predominantly coalesced, nevertheless few regions of uncoalesced ε-Ge regions. A targeted ε-Ge epilayer thickness of 30 nm in the S2-Ge_In‑terminated_ sample would have significantly increased the likelihood of strain relaxation due to In-termination induced agglomeration, which would increase the thickness of the ε-Ge epilayer close to or higher than the critical layer thickness value (45 nm at 1.6% strain^27^). Moreover, the coalesced regions of the ε-Ge epilayer show twin defects appearing to originate from ∼4 nm away from the ε-Ge/In_0.24_Ga_0.76_As heterointerface, as apparent from the HR-TEM image in Figure 8c. However, obtaining the inverse-FFT from the twin defect diffraction spots, (1̅ 1 1̅_t_) and (1̅ 1̅ 1_t_), highlighted in the diffraction pattern (for HR-TEM micrograph of Figure 8c) in Figure 8d, show a different picture altogether.^56,57^ Shown in Figure 8e,f are the inverse-FFT patterns of the twin defects along the planes (1̅ 1 1̅) and (1̅ 1̅ 1), where the lattice lines are in the direction between [1̅ 1 1̅] and [2̅ 0 0] (Figure 8e); between [1̅ 1̅ 1] and [2̅ 0 0] (Figure 8f), however, the twins visibly from the inverse-FFT and the real HR-TEM images are in the (1̅ 1 1̅) and (1̅ 1̅ 1) planes, respectively. The (1̅ 1 1̅_t_) twin defect in Figure 8e is sharp and intense, which shows the origin of the twin defects to be emanating from the interface region itself, rather than ∼4 nm away from the ε-Ge/In_0.24_Ga_0.76_As heterointerface as apparently visible from Figure 8c. The less sharp and less intense (1̅ 1̅ 1_t_) twin defect in Figure 8f, though possibly initiated away from the interface, could also be attributed to originating precisely from the heterointerface region. The amorphous ring observed in Figure 8d represents the glue (bottom left region in Figure 8c) used during the TEM sample preparation. The inverse-FFT patterns, obtained from masking the slip plane diffraction spots, in the inset of Figure 8d shows that the (1̅ 1̅ 1) plane consists of both positive and negative edge dislocations, whereas (1̅ 1 1̅) plane consists of only negative edge dislocations. In both planes, the misfit dislocations originate from the ε-Ge/In_0.24_Ga_0.76_As heterointerface for this S2-Ge_In‑terminated_ sample, unlike the S1-Ge_As‑terminated_ sample in Figure 7. The TEM for the S2-Ge_In‑terminated_ sample shows significant microtwin defects originating at the interface, which are absent in the S1-Ge_As‑terminated_ sample. Defects similar to this have been reported for GaP on a Si heterostructure material system,^58,59^ where despite a small lattice mismatch (∼0.37%) between GaP and the Si substrate, microtwin defects were found to originate right from the gallium-rich heterointerface. Multiple microtwin defects at the GaP/Si heterointerface were attributed to charge buildup, leading to higher interface energy. We believe that this could be the probable cause of an In-rich In_0.24_Ga_0.76_As surface promoting the growth of twins in the S2-Ge_In‑terminated_ sample. These twins are the primary reasons for the partial strain relaxation, shown in Raman spectroscopy above, the poor crystalline quality (inferred from XRD) and the lower carrier lifetime of the ε-Ge epilayer. Effects of these twin defects and the In termination at the heterointerface over the magnetotransport and the band alignment properties are presented in the next sections.

### 3.6 Magnetotransport Properties of Tensile-Strained Ge on As- and In-Terminated InGaAs

The magnetotransport properties of both S1-Ge_As‑terminated_ and S2-Ge_In‑terminated_ samples having As-rich and In-rich Ge/In_0.24_Ga_0.76_As heterointerfaces, respectively, were studied using the van der Pauw configuration.^60,61^ Measurements were performed at 4.2 and 296 K temperatures with a magnetic field (*B*) applied parallel to the growth direction (≤±1.4 T) and with current applied in the plane of the samples. In van der Pauw geometries, symmetrization and antisymmetrization were used to obtain the longitudinal magnetoresistance (i.e., the Hall resistance *R*_*XX*_ (Ω/□)) and the transverse magnetoresistance (i.e., Hall resistance *R*_*XY*_) versus *B*. Type of carriers (n-type or p-type) and carrier concentrations (densities) were obtained from the Hall resistance *R*_*XY*_ vs *B* and the Hall resistance *R*_*XX*_. The slope of *R*_*XY*_ vs *B* allows one to determine the carrier densities, denoted as *N*_e_ for n-type and *N*_h_ for p-type semiconductors. As observed later, quantum interference effects obtained at low temperatures (4.2 K or lower) can lead to magnetoresistance; however, quantum interference disappears at higher temperatures. As noted from Figure 9a at both 296 and 4.2 K, and from Figure 9b at 296 K, *R*_*XY*_ shows a linear dependence on *B* in the range ±1.4 T. Further, at 296 K, no *R*_*XX*_ was observed for either heterostructure as a function of *B*. The linear dependences of *R*_*XY*_ on *B* and *R*_*XX*_ as a function of *B* (only at low temperature), shown in Figure 10, but not at room temperature, indicate that electrical transport is dominated by single carriers in both S1-Ge_As‑terminated_ and S2-Ge_In‑terminated_ heterostructures.^60,61^

**Figure 9:**
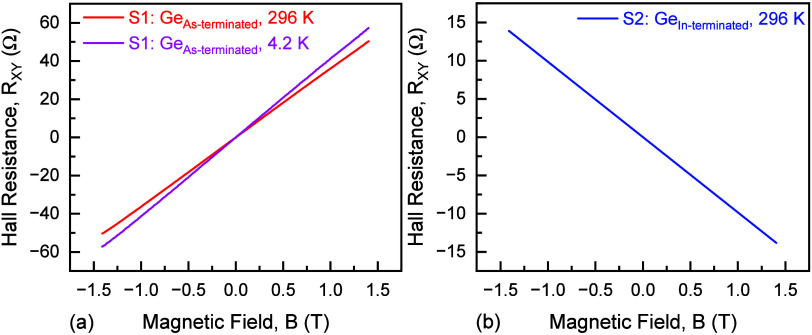
Hall resistance, *R*_*XY*_ (transverse magnetoresistance) as a function of magnetic field, and *B* over a range of (≤±1.4 T) for (a) S1–Ge_As‑terminated_ and (b) S2–Ge_In‑terminated_ samples. Both curves exhibit linear dependence on *B*, indicative of single-carrier behavior (positive slope defined as n-type conduction, negative slope as p-type conduction).

**Figure 10:**
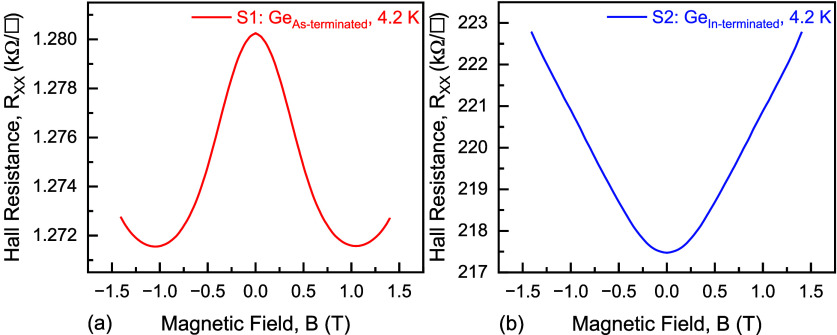
Measured Hall resistance *R*_*XX*_ (longitudinal magnetoresistance) as a function of magnetic field *B* (≤±1.4 T) at 4.2 K for the (a) S1-Ge_As‑terminated_ and (b) S2-Ge_In‑terminated_ samples.

The type of carriers and corresponding densities were extracted using the single-carrier transport model,^60,61^ as summarized in Table 1. The signs of the slopes in Figure 9a,b and the table are defined in such a way that a positive slope indicates electron conduction (n-type), while a negative slope indicates hole conduction (p-type). The ε-Ge epilayer is determined to be n-type (p-type) when grown on As-terminated (In-terminated) constant composition In_0.24_Ga_0.76_As buffer. In S2-Ge_In‑terminated_, the observation of unexpected p-type behavior is due to In dangling at the interface. These dangling bonds form when In atoms at the interface have fewer neighboring atoms than in the bulk layer, resulting in incomplete bonding and the emergence of localized electronic states within the Ge bandgap.^62^ Notably, these interface-induced states often lie near the valence band edge of Ge, acting as acceptor-like traps that can pin the Fermi level closer to the valence band. This Fermi level pinning leads to upward band bending and hole accumulation at the ε-Ge/In_0.24_Ga_0.76_As interface, effectively inducing the observed p-type conduction.

**Table 1:** Summary of the Magnetotransport Properties of the ε-Ge Epilayer in Samples S1-Ge_As‑terminated_ and S2-Ge_In‑terminated_ Grown on Constant Composition In_0.24_Ga_0.76_As Buffer Layers with As- and In-Termination

		density (cm^–3^)		mobility (cm^2^/(V s))		scattering rate (fs)	
In_0.24_Ga_0.76_As surface termination	carrier type	4.2 K	296 K	4.2 K	296 K	4.2 K	296 K
As-atom	electrons	5.33 × 10^18^	5.77 × 10^18^	321	317	21.9	21.7
In-atom	holes		2.54 × 10^19^		3.34		0.228

The electron density of S1-Ge_As‑terminated_ is *N*_e_ = 5.33 × 10^18^ cm^–3^ at 4.2 K, representing a small decline from *N*_e_ = 5.77 × 10^18^ cm^–3^ at 296 K. This small difference between 4.2 and 296 K indicates a degenerate doping occurring during the growth of the (unintentionally doped) heterostructure, attributed to 1.6% tensile strain in the ε-Ge epilayer. In the case of S2-Ge_In‑terminated_ at 296 K, the hole density *N*_h_ = 2.54 × 10^19^ cm^–3^ is more than 4× the electron density in the S1-Ge_As‑terminated_ at 296 K. At 4.2 K, the S2-Ge_In‑terminated_ sample displayed very high resistivity, which impeded the precise determination of the slope of *R*_*XY*_ vs *B* and hence of a determination of *N*_h_ at 4.2 K. The high resistivity of the S2-Ge_In‑terminated_ sample is supported by the *R*_*XX*_ plot shown in Figure 10b, whereas, at 296 K, the S1-Ge_As‑terminated_ sample exhibited negligible *R*_*XX*_ as mentioned; however, at 4.2 K, a small negative magnetoresistance emerges, shown in Figure 10a, characterized by a sharp peak near *B* = 0. These magnetoresistance characteristics indicate the presence of the weak localization (WL) effect,^63–67^ which supports our earlier assertion of a heavily doped (and hence disordered) carrier system. Weak localization is a low-temperature quantum interference phenomenon occurring in disordered conductive systems, characterized by an enhancement of electrical resistance in the absence of an external magnetic field.^63–67^ The WL arises from the constructive quantum interference of electrons or holes on pairs of time-reversed closed-path electron trajectories (under negligible spin–orbit coupling). The closed-path trajectories are set up by scattering events on impurities in a disordered system. A defining experimental feature of WL is the observation of negative magnetoresistance at low *B*, wherein the application of *B* suppresses constructive quantum interference between electron paths, resulting in a reduction of resistance, and thus, increasing *B* decreases the resistivity. The occurrence of WL is for disordered systems at low temperatures, where quantum interference effects appear. This behavior was evident in Figure 10a, which shows an initial decrease in resistance with increasing *B*.^65,66^

At 4.2 K, the S2-Ge_In‑terminated_ sample exhibits a weak positive magnetoresistance, as shown in Figure 10b, incompatible with WL. A weak positive magnetoresistance indicates multicarrier transport; however, the transport in this sample is hole driven. The multicarrier signature hence suggests the existence of two-hole populations in the ε-Ge layer, possibly at different distances from the interface, with one population strongly dominating transport properties. This could be attributed to the contribution from the uncoalesced regions of ε-Ge, where holes from the In_0.24_Ga_0.76_As layers underneath offer conduction, as further investigated from the experimentally constructed band alignment in the next section. We calculate the mobility to be 317 cm^2^/(V s) for S1-Ge_As‑terminated_ at 296 K and 321 cm^2^/(V s) at 4.2 K. The slight increase in mobility at low temperatures is again indicative of transport in a heavily doped system, where ionized impurity scattering strongly dominates phonon scattering. The S2-Ge_In‑terminated_ sample exhibited a very high resistivity at 4.2 K, preventing the determination of carrier density and mobility. Nevertheless, at 296 K, the S2-Ge_In‑terminated_ sample showed a mobility of 3.34 cm^2^/(V s), which is 100× lower than that of the S1-Ge_As‑terminated_ sample. This lower mobility is primarily due to the higher effective mass of holes compared to electrons in p-type Ge, since the heavier holes experience stronger scattering than electrons.

Panels a and b of Figure 11 depict the measured *R*_*XX*_ at *B* = 0 vs temperature for the S1-Ge_As‑terminated_ and S2-Ge_In‑terminated_ heterostructures, respectively. The *R*_*XX*_ in S1-Ge_As‑terminated_ shows a mild temperature dependence, and as the temperature decreases from 292 K, the carrier density decreases and, concomitantly, the screening is reduced. This leads to increased impurity and phonon scattering, contributing to higher *R*_*XX*_. Around 175 K, a downturn in *R*_*XX*_ is observed, attributed to the freezeout of phonons, which reduces phonon scattering. However, below approximately 35 K, an upturn in resistance appears again, attributed to WL. The dependence on temperature of *R*_*XX*_ in S2-Ge_In‑terminated_ can be explained in terms of Mott variable-range hopping (VRH), which characterizes low-temperature resistivity in disordered systems with localized electronic states.^68–70^ As temperature decreases, *R*_*XX*_ increases exponentially due to the reduced probability of thermally activated hopping between localized states, resulting in suppressed conductivity.^69,70^ The functional dependence of *R*_*XX*_ on the temperature expected from VRH is consistent with the results in Figure 11b. Furthermore, the next section presents an investigation of the band alignment, identifying a probable reason for the observation of two-hole populations in the S2-Ge_In‑terminated_ sample and confinement of electrons in the S1-Ge_As‑terminated_ sample.

**Figure 11:**
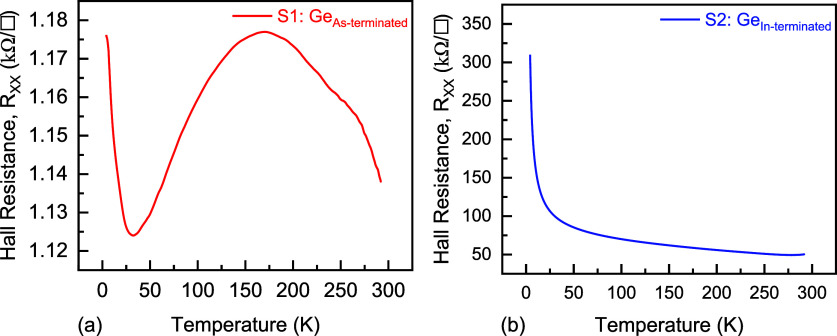
Hall resistance *R*_*XX*_ at zero magnetic field (*B* = 0 T) vs temperature for the (a) S1-Ge_As‑terminated_ and (b) S2-Ge_In‑terminated_ samples.

### 3.7 Effect of In/As Termination on Energy Band Offset Determined via XPS

Construction of the energy band alignment across the ε-Ge on the In_0.24_Ga_0.76_As heterostructure is essential to understand the optical and electrical carrier confinement within the ε-Ge active layer of interest. Therefore, the valence band offsets at the ε-Ge/In_0.24_Ga_0.76_As heterointerface were determined using the XPS technique, where the atomic CL binding energies of (i) top ε-Ge (Ge 3d) epilayer; (ii) ε-Ge/In_0.24_Ga_0.76_As heterointerface region (Ge 3d, As 3d, and In 4d); and (iii) bottom In_0.24_Ga_0.76_As buffer layer (As 3d and In 4d) were measured along with the VBM of the respective materials in the corresponding layers. To study the effect of the type of surface termination (atop the In_0.24_Ga_0.76_As layer) on the band offsets at the ε-Ge/In_0.24_Ga_0.76_As heterointerface, two samples S1-Ge_As‑terminated_ and S2-Ge_In‑terminated_ were synthesized, where (i) 30 nm (25 nm) thick ε-Ge grown on As-terminated (In-terminated) In_0.24_Ga_0.76_As was utilized to measure the CL binding energies and VBM of ε-Ge; (ii) CL binding energies at the heterointerface region were acquired through sputtering the top ε-Ge epilayer by Ar^+^ ion to arrive at the ε-Ge/In_0.24_Ga_0.76_As interface region; and (iii) further sputtering was used to collect the CL binding energies and VBM from the 0.75 μm (0.35 μm) thick constant composition In_0.24_Ga_0.76_As stressor layer. All of the elemental CL binding energy distribution curves and the VBM were shift-corrected using the standard peak position of the adventitious carbon, C 1s CL, at 285 eV.^40^ It is imperative to note that the native oxide from the top surface of each sample was removed along with the adventitious carbon by Ar^+^ ion sputtering prior to the XPS spectral acquisition from the top ε-Ge layer. During the entire XPS spectral acquisition process, a low-energy (0 to 10 eV) electron gun was used to neutralize the positive charges accumulated in the samples due to the loss of electrons during photoemission. The valence band offset Δ*E*_V_ was determined using the standard procedure of Kraut’s method,^41^ widely employed to experimentally construct the band alignment at semiconductor heterointerfaces and semiconductor–dielectric heterojunctions. Here, binding energy peak positions of spin–orbit split Ge 3d {(*E*_Ge3d_5/2__, *E*_Ge3d_3/2__)^ε‑Ge^} and As 3d {(*E*_As3d_5/2__, *E*_As3d_3/2__)^InGaAs^} were extracted from the S1-Ge_As‑terminated_ sample, as shown in Figure 12. Using these, the valence band offset Δ*E*_V,As‑terminated_ can be expressed as^41^

$$\Delta E_{V,\mathrm{As‐terminated}}=\left(E_{\mathrm{Ge}3d_{5/2}}^{\varepsilon \mathrm{‐Ge}}-E_{\mathrm{VBM}}^{\varepsilon \mathrm{‐Ge}}\right)-\left(E_{\mathrm{As}3d_{5/2}}^{\mathrm{InGaAs}}-E_{\mathrm{VBM}}^{\mathrm{InGaAs}}\right)+\left(E_{\mathrm{As}3d_{5/2}}^{\mathrm{InGaAs}}\left(i\right)-E_{\mathrm{Ge}3d_{5/2}}^{\varepsilon \mathrm{‐Ge}}\left(i\right)\right)$$

where $\left(E_{\mathrm{Ge}3d_{5/2}}^{\varepsilon \mathrm{‐Ge}}-E_{\mathrm{VBM}}^{\varepsilon \mathrm{‐Ge}}\right)$ is the binding energy separation between the Ge 3d_5/2_ CL and VBM of the ε-Ge epilayer and $\left(E_{\mathrm{As}3d_{5/2}}^{\mathrm{InGaAs}}-E_{\mathrm{VBM}}^{\mathrm{InGaAs}}\right)$ is of As 3d_5/2_ CL and VBM from the In_0.24_Ga_0.76_As layer in the S1-Ge_As‑terminated_ sample, and the last term $\left(E_{\mathrm{As}3d_{5/2}}^{\mathrm{InGaAs}}\left(i\right)-E_{\mathrm{Ge}3d_{5/2}}^{\varepsilon \mathrm{‐Ge}}\left(i\right)\right)$ is the binding energy separation between As 3d_5/2_ CL and Ge 3d_5/2_ CL at the ε-Ge/In_0.24_Ga_0.76_As heterointerface of the S1-Ge_As‑terminated_ sample. The values of these three terms in the order of their occurrence in the equation are 28.99 ± 0.05, 40.56 ± 0.05, and 11.79 ± 0.05 eV, respectively. The valence band offset Δ*E*_V,As‑terminated_ was extracted to be 0.22 ± 0.1 eV.

**Figure 12:**
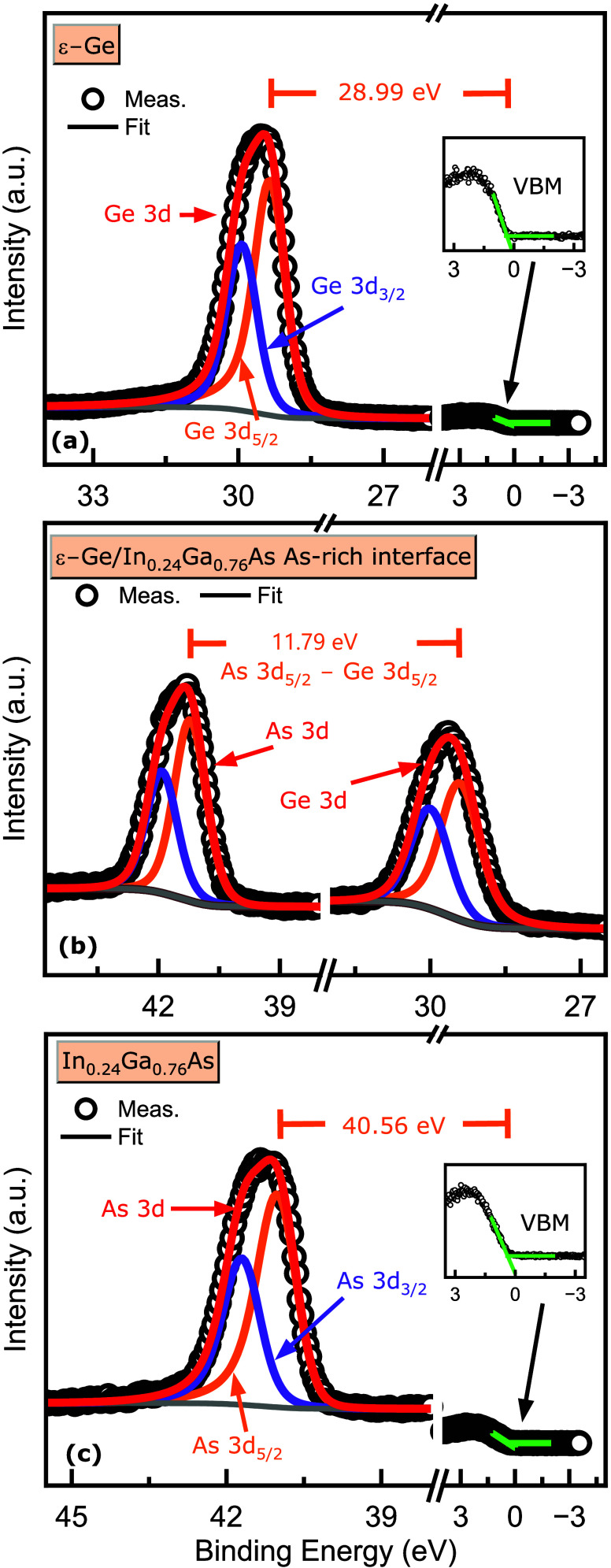
X-ray photoelectron spectra recorded for the valence band analysis of ε-Ge/In_0.24_Ga_0.76_As heterostructure of S1–Ge_As‑terminated_ sample having As-terminated In_0.24_Ga_0.76_As stressor with core level binding energy shifts from (a) 30 nm thick ε-Ge epilayer (b) As-rich ε-Ge/In_0.24_Ga_0.76_As heterointerface, and (c) bulk-like constant composition In_0.24_Ga_0.76_As buffer layer.

Similarly, binding energy peak positions of spin–orbit split Ge 3d {(*E*_Ge 3d5/2_, E_Ge3d_3/2__)^ε‑Ge^} and In 4d {(*E*_In4d_)^InGaAs^} were extracted from the S2-Ge_In‑terminated_ sample as shown in Figure 13, where the valence band offset, Δ*E*_V,In‑terminated_, is expressed as^41^

$$\Delta E_{V,\mathrm{In‐terminated}}=\left(E_{\mathrm{Ge}3d_{5/2}}^{\varepsilon \mathrm{‐Ge}}-E_{\mathrm{VBM}}^{\varepsilon \mathrm{‐Ge}}\right)-\left(E_{\mathrm{In}4d}^{\mathrm{InGaAs}}-E_{\mathrm{VBM}}^{\mathrm{InGaAs}}\right)+\left(E_{\mathrm{In}4d}^{\mathrm{InGaAs}}\left(i\right)-E_{\mathrm{Ge}3d_{5/2}}^{\varepsilon \mathrm{‐Ge}}\left(i\right)\right)$$

where $\left(E_{\mathrm{Ge}3d_{5/2}}^{\varepsilon \mathrm{‐Ge}}-E_{\mathrm{VBM}}^{\varepsilon \mathrm{‐Ge}}\right)$ is the binding energy separation between the Ge 3d_5/2_ CL and VBM of the ε-Ge epilayer, (*E*_In4d_^InGaAs^ – *E*_VBM_^InGaAs^) of In 4d and VBM from the In_0.24_Ga_0.76_As layer, and the last term $\left(E_{\mathrm{In}4d}^{\mathrm{InGaAs}}\left(i\right)-E_{\mathrm{Ge}3d_{5/2}}^{\varepsilon \mathrm{‐Ge}}\left(i\right)\right)$ is the binding energy separation between In 4d CL and Ge 3d_5/2_ CL at the ε-Ge/In_0.24_Ga_0.76_As heterointerface of the S2-Ge_In‑terminated_ sample. The values of these three terms in the order of their occurrence in the equation are 29.07 ± 0.05, 17.21 ± 0.05, and −11.88 ± 0.05 eV, respectively, and Δ*E*_V,In‑terminated_ was extracted to be −0.02 ± 0.1 eV. Also, even considering the As 3d_5/2_ CL in the S2-Ge_In‑terminated_ sample, Δ*E*_V,In‑terminated_ was evaluated to be 0.07 ± 0.1 eV, which is still 0.15 eV lower than Δ*E*_V,As‑terminated_. The schematic band alignment as constructed using the XPS analysis is shown in Figure 14. It is evident that both the electrons and holes are confined within the ε-Ge epilayer of the S1-Ge_As‑terminated_ sample, with type-I energy band alignment, shown in Figure 14a, using the As-rich interface engineering. It was reported previously^12^ that the band alignment for a 1.6% ε-Ge on As-terminated In_0.24_Ga_0.76_As stressor without In overshoot in the In_*x*_Ga_1–*x*_As LGB design was also experimentally determined as type-I with Δ*E*_V_ = 0.35 eV.^12^ However, with the In-rich interface of the S2-Ge_In‑terminated_ sample, the ε-Ge epilayer confines only the electrons in the type-II energy band alignment, seen in Figure 14b, and the holes from the ε-Ge epilayer leak to the In_0.24_Ga_0.76_As buffer underneath.

**Figure 13:**
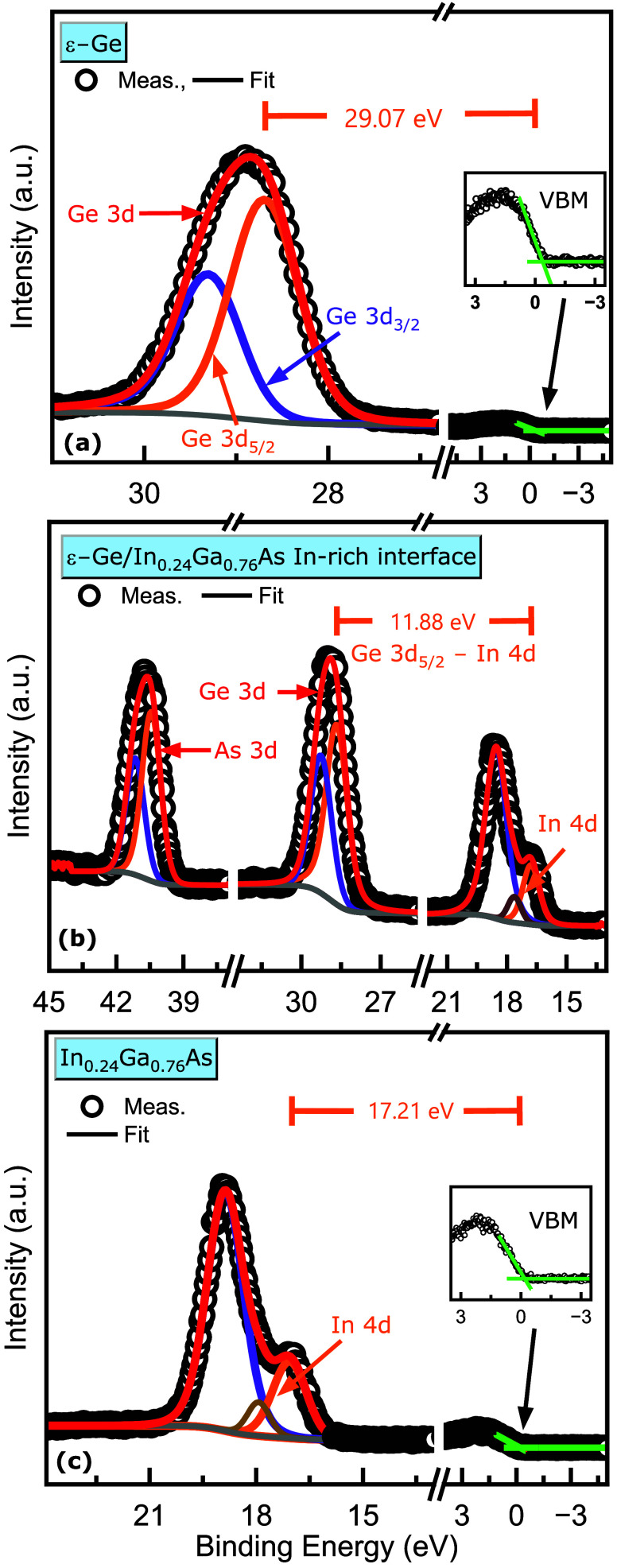
X-ray photoelectron spectra used in the valence band analysis of ε-Ge/In_0.24_Ga_0.76_As heterostructure of S2-Ge_In‑terminated_ sample having In-terminated In_0.24_Ga_0.76_As stressor with core level binding energy shifts from (a) 25 nm thick ε-Ge epilayer, (b) In-rich ε-Ge/In_0.24_Ga_0.76_As heterointerface, and (c) bulk-like constant composition In_0.24_Ga_0.76_As buffer layer.

**Figure 14:**
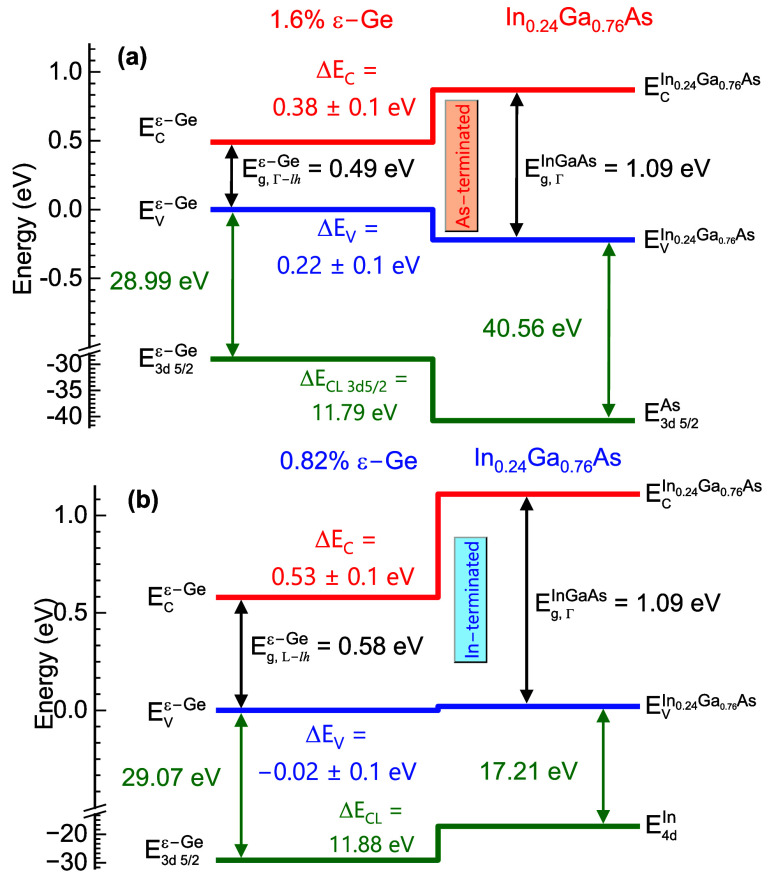
Schematic energy band alignment of the ε-Ge/In_0.24_Ga_0.76_As heterostructures: (a) S1-Ge_As‑terminated_ having As-rich heterointerface forming type-I energy band alignment with Δ*E*_V_ = 0.22 ± 0.1 eV and Δ*E*_C_ = 0.38 ± 0.1 eV, confining both electrons and holes in the ε-Ge epilayer; (b) S2-Ge_In‑terminated_ having In-rich heterointerface forms type-II energy band alignment with Δ*E*_V_ = −0.02 ± 0.1 eV and Δ*E*_C_ = 0.53 ± 0.1 eV confining electrons in ε-Ge and leaking holes to the In_0.24_Ga_0.76_As layer.

This experimental result differs from the theoretically calculated band alignment types of ε-Ge/In_0.30_Ga_0.70_As heterostructure material system by Pavarelli et al.,^25^ where energy band alignment was obtained as type-I for 100% Ge bonded with group-III atoms (Δ*E*_V_ = 0.51 eV) and type-II for 100% Ge bonded with group-V atoms (Δ*E*_V_ = −0.01 eV) at abrupt heterointerfaces, with no charges balanced. The band offsets for the intermediate stoichiometries were interpolated, where at around 52% Ge-group-III and 48% Ge-group-V bonds, the band alignment transitioned from type-II to type-I with Δ*E*_V_ = 0 eV, further indicating the band alignment was type-II for the group-V atoms rich ε-Ge/In_0.30_Ga_0.70_As interface and type-I for the group-III atoms rich ε-Ge/In_0.30_Ga_0.70_As interface. There were no defects incorporated into the atomic models used for these calculations nor were the atomic interdiffusion models incorporated for maintaining charge neutrality at the interfaces. Similarly, first-principles calculations were carried out by Greene- Diniz and Grüning for the ε-Ge/In_0.25_Al_0.75_As heterostructure material system^19^ and all calculations therein involved charge-balanced heterointerfaces. Their results showed type-I band alignment for both group-III terminated (Δ*E*_V_ = 1.35 eV) and group-V terminated (Δ*E*_V_ = 0.86 eV) abrupt interfaces (0 ML interdiffusion) of the ε-Ge/In_0.25_Al_0.75_As heterostructure. However, when atomic interdiffusion of group-III atoms into 1 ML of Ge was modeled, the band alignment transitioned from type-I to type-II with Δ*E*_V_ = 1.61 eV, but for interdiffusion of group-V atoms into 2 MLs of Ge it stayed as type-I with Δ*E*_V_ = 0.22 eV. Experimentally, the APT results of ε-Ge/In_0.24_Ga_0.76_As^26^ and Ge/AlAs^39^ heterostructures (where both III–V buffer layers were As-terminated and synthesized in the same MBE chamber as the present work) showed arsenic up-diffusion of ∼6 Å (2 MLs) into ε-Ge and Ge epilayers, respectively, with corresponding type-I band offsets of Δ*E*_V_ = 0.35 eV^12,26^ and Δ*E*_V_ = 0.75 eV.^39^ Another experimentally determined band alignment for As-rich ε-Ge/In_0.26_Al_0.74_As heterointerface was of type-I with Δ*E*_V_ = 0.56 eV.^23^ No experimental study was found on the group-III terminated InGa­(or Al)As buffer layer that acted as a stressor template to synthesize a ε-Ge epilayer. Moreover, all of the experimental studies shown here with As-terminated III–V buffers had type-I band alignment, necessary for lasers and photodetectors. Hence, it is crucial to engineer the interface conditions while synthesizing a ε-Ge/In_0.24_Ga_0.76_As heterostructure such that the interface is As-rich, rather than In- or group-III-rich, to realize a device-quality material system beneficial to fabricate exceptional group-IV-based optoelectronic and photonic devices.

## 4 Conclusions

Tensile-strained Ge epitaxial layers were synthesized on constant composition In_0.24_Ga_0.76_As buffer layers with two types of surface terminations—arsenic and indium—using solid source molecular beam epitaxy. The ε-Ge epilayer was fully strained at 1.6% in the Ge_As‑terminated_ sample, whereas it partially relaxed to 0.82% in the Ge_In‑terminated_ sample, determined using X-ray and Raman spectroscopy analyses. Further investigation using cross-sectional transmission electron microscopy analysis revealed an abrupt and coherent ε-Ge/In_0.24_Ga_0.76_As heterointerface in the Ge_As‑terminated_ sample, whereas microtwin defects nucleated in the Ge_In‑terminated_ sample right from the ε-Ge/In_0.24_Ga_0.76_As heterointerface, promoting strain relaxation of the ε-Ge epilayer. The resulting impact on the quality of the ε-Ge epilayer was examined by extracting a high-minority carrier lifetime from the Ge_As‑terminated_ (525 ns) sample compared to the Ge_In‑terminated_ (69 ns) sample using the microwave-reflection photoconductive decay technique. Moreover, the Ge_As‑terminated_ sample exhibited a higher electron mobility of 321 cm^2^/(V s) than the Ge_In‑terminated_ sample, which had a low hole mobility of 3.34 cm^2^/(V s). The ε-Ge epilayer in these two heterostructures exhibited a single-carrier transport. However, the magnetotransport properties showed two-hole contributions in the Ge_In‑terminated_ sample, which were further corroborated by the experimentally determined energy band alignments using X-ray photoelectron spectroscopy, where the type-I energy band alignment in the Ge_As‑terminated_ sample showed superior carrier confinement (Δ*E*_V_ = 0.22 eV and Δ*E*_C_ = 0.38 eV) and the type-II in the Ge_In‑terminated_ sample showed poor carrier confinement (Δ*E*_V_ = – 0.02 eV and Δ*E*_C_ = 0.53 eV) within the ε-Ge epilayers. Therefore, engineering the ε-Ge/In_0.24_Ga_0.76_As heterointerface with precise surface termination of the underlying InGaAs stressor layer during epitaxy, preferably arsenic atoms, offers several advantages for realizing efficient group-IV-based optoelectronic and photonic devices.
